# Re-examining the drivers of variation in parasite loads across hosts in the Tallis-Leyton model

**DOI:** 10.1007/s00285-025-02241-w

**Published:** 2025-07-26

**Authors:** R. McVinish

**Affiliations:** School of Mathematics and Physics, University of Queensland, Brisbane, Australia

**Keywords:** Aggregation, Convex order, Gini index, Lorenz order, Negative binomial distribution, Pietra index, 60E15, 92D25, 60K25

## Abstract

The Tallis-Leyton model is a simple model of parasite acquisition where parasites accumulate in the host without affecting the host’s mortality, or eliciting any immune reaction from the host. Furthermore, the parasites do not reproduce in the host. We examine how the variability in parasite loads among hosts is affected by the rate of infectious contacts, the distribution of parasite entering the host during infectious contacts, the host’s age, and the distribution of parasite lifetimes. Motivated by empirical studies in parasitology, variability is examined in the sense of the Lorenz order and related metrics. Perhaps counterintuitively, increased variability in the distribution of parasite lifetimes is seen to decrease variability in the parasite loads among hosts.

## Introduction

The distribution of parasites among their host population typically displays a high degree of variation; some hosts are infected with many parasites while many hosts have comparatively few. This phenomenon is almost universally observed in wild populations (Shaw and Dobson 1995; Poulin 2007). Following the usual practice in the parasitology literature, we call this phenomenon *aggregation* (Pielou 1977; Wilson et al. 2001; Poulin 2011).

Unfortunately, there is no universally accepted measure of aggregation. Instead, different authors use different metrics of aggregation to summarise the parasite’s distribution (Pielou 1977; McVinish and Lester 2020; Morrill et al. 2023). Despite claims that these measures have identical interpretations and more-or-less predict each other (Reiczigel et al. 2014), different methods can give opposing answers (McVinish and Lester 2020, Figure 1). The most commonly used measures of aggregation in theoretical models are the variance-to-mean ratio (Isham 1995; Barbour and Pugliese 2000; Herbert and Isham 2000; Peacock et al. 2018) and the *k* parameter of the negative binomial distribution (Anderson and May 1978a, b; Rosà and Pugliese 2002; Schreiber 2006; McPherson et al. 2012), where the negative binomial distribution has mean *m* and variance $$ m + m^{2}/k$$. Both of these measures can be interpreted as quantifying how over dispersed the distribution of parasite load is relative to a Poisson distribution.

An alternative view of aggregation was put forward by Poulin (1993), arguing that a measure of the discrepancy between the observed distribution of parasites in the hosts and the ideal distribution where all hosts are infected with the same number of parasites would be the best measure of aggregation. This view puts the Lorenz ordering of distributions (Lorenz 1905; Arnold and Sarabia 2018) central in the study of aggregation. While the coefficient of variation is perhaps the most widely known measure having a direct relationship to the Lorenz order (Arnold and Sarabia 2018, Sections 5.2.1 & 5.4), Poulin (1993) proposed using a different index, *D*, as a measure of this discrepancy. It can be shown that, up to a factor that goes to one as the sample size increases, Poulin’s *D* is Gini’s concentration ratio (Gini 1914, 2005), also known as the estimator of the Gini index (Arnold and Sarabia 2018, Equation 5.85). Poulin’s *D* has since become one of the standard measures of aggregation used in studies of wild parasite populations (Herrero-Cófreces et al. 2021; Rodríguez-Hernández et al. 2021; Schrock et al. 2025).

The aim of this paper is to characterize how different processes in the Tallis-Leyton model (Tallis and Leyton 1969) shape parasite aggregation in the sense of the Lorenz ordering and related indices. Only population values of these indices are considered, rather than their estimators. Despite the growing importance of the Lorenz order in empirical studies of parasite distributions following Poulin’s proposal, we are unaware of any other theoretical study of parasite acquisition using the Lorenz order. In Sect. 2 we review some background on the Lorenz ordering and the closely related convex ordering of distributions. The Tallis-Leyton model is analysed in Sect. 3. We first show show how the host’s parasite load can be represented as a compound Poisson distribution. This representation is then applied to determine how each of the model parameters affect the Lorenz ordering of the distribution of parasites in the host. The final part of the analysis shows that the host’s parasite load is asymptotically normally distributed in the limit as the rate of infectious contacts goes to infinity. This allows the indices to be approximated in terms of the mean and variance. The paper concludes with a discussion of future challenges in analysing models of parasite aggregation.

## Background

### Tallis-Leyton model

Tallis and Leyton (1969) proposed the following model for the parasite load *M*(*a*) of a definitive host at age *a*, conditional on survival of the host to age *a*. The host is parasite free at birth so $M(0) = 0$. During its lifetime, the host makes infectious contacts following a Poisson process with constant rate $\lambda$. At each infectious contact, a random number of parasites *N* enter the host. Once a parasite enters the host, it survives for a random period of time *T*. The lifetimes of parasites, numbers of parasites entering the host at infectious contacts, and the Poisson process of infectious contacts are all independent. The parasites are assumed to have no effect on the host mortality so the host’s parasite load at age *a* is independent of the host surviving to age *a*. Henceforth, we will simply refer to *M*(*a*) as the parasite load of a host age *a*. Although we won’t make use of this fact, we note that this process also describes an infinite server queue with bulk arrivals and general independent service times (Holman et al. 1983).

Let $G_X$, $F_X$ and $\bar{F}_X = 1 - F_{X}$ denote the probability generating function (PGF), distribution function, and survival function of a random variable *X*. We write $G_{M}(\cdot;a)$ for the PGF of *M*(*a*). Tallis and Leyton (1969) showed

1 $$\begin{aligned} G_{M}(z;a) = \exp \left( \lambda \int _{0}^{a} \left[ G_{N}(1 + \bar{F}_{T}(a-s) (z-1)) -1 \right] ds\right). \end{aligned}$$

Using the well known relationship between the moments of a random variable and derivatives of its PGF at zero, we see that the mean and variance of *M*(*a*) are

2 $$\begin{aligned} \mu (a)&= \lambda \mathbb {E}\left[ N\right] \int _{0}^{a} \bar{F}_{T}(s)\, ds \end{aligned}$$

3 $$\begin{aligned} \sigma ^{2}(a)&= \lambda \mathbb {E} \left[ N(N-1)\right] \int _{0}^{a} \bar{F}_{T}^2(s)\, ds + \lambda \mathbb {E} \left[ N\right] \int _{0}^{a} \bar{F}_{T}(s)\, ds. \end{aligned}$$

Hence, the variance-to-mean ratio is

4 $$\begin{aligned} \text {VMR}(M(a)) = 1 + \frac{\mathbb {E} \left[ N(N-1) \right] }{\mathbb {E}\left[ N\right] } \frac{\int ^a_0 \bar{F}^{2}_{T}(s) ds}{\int ^a_0 \bar{F}_{T}(s) ds}. \end{aligned}$$

Assuming $\mathbb {E} \left[ N\right] < \infty$ and $\mathbb {E} \left[ T\right] < \infty$, the limiting distribution of parasite load as $a\rightarrow \infty$ exists and has PGF

5 $$\begin{aligned} G_{M}(z,\infty ) = \exp \left( \lambda \int _{0}^{\infty } \left[ G_{N}(1 + \bar{F}_{T}(s) (z-1)) -1 \right] ds\right). \end{aligned}$$

An appropriate rescaling of the host age, rate of infectious contacts, and parasite lifetimes leaves the distribution of the host’s parasite load unchanged. Specifically, for any $c>0$ let $\tilde{M}(a)$ represent the parasite load of host age *a* in the Tallis-Leyton model with parameters $\tilde{\lambda } = \lambda c$, $\tilde{N} {\mathop {=}\limits ^{d}} N$ and $\tilde{T} {\mathop {=}\limits ^{d}} T/c$. Then $M(ca) {\mathop {=}\limits ^{d}} \tilde{M}(a)$. To see this, apply the change of variable $s=cr$ in the integral in (1) for the PGF of *M*(*ca*):

6 $$\begin{aligned} G_{M}(z;ca)&= \exp \left( \lambda \int _{0}^{ca} \left[ G_{N}(1 + \bar{F}_{T}(ca-s) (z-1)) -1 \right] ds\right) \end{aligned}$$

7 $$\begin{aligned}&= \exp \left( \lambda c \int _{0}^{a} \left[ G_{N}(1 + \bar{F}_{T}(ca-cr) (z-1)) -1 \right] dr\right). \end{aligned}$$

Upon noting $\bar{F}_{\tilde{T}} (s) = \bar{F}_{T}(cs)$, it follows that $G_{M}(z;ca) = G_{\tilde{M}}(z;a)$.

### Convex order and Lorenz order

Lorenz (1905) proposed the Lorenz curve as a graphical measure of inequality. The following general definition of the Lorenz curve was given by Gastwirth (1971).

#### Definition

The *Lorenz curve *$L: [0,1] \rightarrow [0,1]$ for the distribution *F* with finite mean $\mu$ is given by

8 $$\begin{aligned} L(u) = \frac{\int _{0}^{u} F^{-1}(y)\, dy}{\mu }, \end{aligned}$$

where $$ F^{-1}$$ is the quantile function

9 $$\begin{aligned} F^{-1}(y) = \sup \{ x: F(x) \le y \} \quad \text {for } y \in (0,1). \end{aligned}$$

Adapting the description in Arnold and Sarabia (2018, Section 3.1) to a parasitology context, the Lorenz curve *L*(*u*) represents the proportion of the parasite population infecting the least infected *u* proportion of the host population. When all hosts are infected with the same number of parasites, the Lorenz curve is given by $L(u) = u$ and is called the egalitarian line. The Lorenz curve never rises above the egalitarian line, that is $L(u) \le u$ for all $u \in [0,1]$.

The Lorenz curve defines a partial order on the class of all distributions on $[0,\infty )$ with finite mean (Arnold and Sarabia 2018, Definition 3.2.1).

#### Definition

Let *X* and *Y* be random variables with the respective Lorenz curves denoted $$ L_{X} $$ and $$ L_{Y}$$. We say *X* is smaller in the *Lorenz order*, denoted $$ X \le _{\mathrm{{Lorenz}}} Y $$ if $$ L_{X}(u) \ge L_{Y}(u)$$ for every $$ u \in [0,1]$$.

The negative binomial distribution, which is extensively used in parasitology, can be compared in the Lorenz order (McVinish and Lester 2024). Specifically, let ${\mathsf{NB}}(m,k)$ denote the negative binomial distribution with mean *m* and variance $m + m^{2}/k$. Then

(i) for any $$ k >0 $$ and $$ 0< m_{1} < m_{2}$$, $${\mathsf{NB}}(m_{2},k) \le _{\mathrm{Lorenz}} {\mathsf{NB}}( m_{1},k)$$, and
(ii) for any $m>0$ and $0< k_{1} < k_{2}$, ${\mathsf{NB}}(m, k_{2}) \le _{\mathrm{Lorenz}} {\mathsf{NB}}(m, k_{1})$.

Closely related to the Lorenz order is the convex order of distributions.

#### Definition

Let *X* and *Y* be two random variables such that $\mathbb {E} \left[ X\right] = \mathbb {E} \left[ Y\right]$. We say *X* is smaller than *Y* in the *convex order*, denoted $X \le _{\mathrm{cx}} Y$, if $\mathbb {E} \left[ \phi (X)\right] \le \mathbb {E} \left[ \phi (Y)\right]$ for all convex functions $\phi: \mathbb {R} \rightarrow \mathbb {R}$, provided the expectations exist.

These two orderings are related since $X \le _{\mathrm{Lorenz}} Y$ if and only if

10 $$\begin{aligned} \mathbb {E}\, \left[ \phi \left( \frac{X}{\mathbb {E}\left[ X\right] }\right) \right] \le \mathbb {E}\, \left[ \phi \left( \frac{Y}{\mathbb {E}\left[ Y\right] }\right) \right] \end{aligned}$$

for every continuous convex function $$\phi\ $$ (Arnold and Sarabia 2018, Corollary 3.2.1). In other words,

11 $$\begin{aligned} X&\le _{\mathrm{Lorenz}} Y&\text {is equivalent to} & \frac{X}{\mathbb {E} \left[ X\right] }&\le _{\mathrm{cx}} \frac{Y}{\mathbb {E} \left[ Y\right] }. \end{aligned}$$

Shaked and Shanthikumar (2007, Section 3.A) provide an extensive review of results on the convex order. We briefly mention some of the important results that are used in our analysis.

- The convex order is *closed under weak limits* provided the expectations also converge (Shaked and Shanthikumar 2007, Theorem 3.A.12 (c)).
- The convex order is *closed under mixtures* (Shaked and Shanthikumar 2007, Theorem 3.A.12 (b)). Let *X*, *Y*, and $\Theta$ be random variables and write $[X \mid \Theta = \theta ]$ and $[Y \mid \Theta = \theta ]$ for the conditional distributions of *X* and *Y* given $\Theta = \theta$. If $[X \mid \Theta = \theta ] \le _{\mathrm{cx}} [Y \mid \Theta = \theta ]$ for all $\theta$ in the support of $\Theta$, then $X \le _{\mathrm{cx}} Y$. As an application of this property we can say that if $X \le _{\mathrm{cx}} Y$ and *Z* is an independent non-negative random variable, then $ZX \le _{\mathrm{cx}} ZY$.
- The convex order is *closed under convolutions* (Shaked and Shanthikumar 2007, Theorem 3.A.12 (d)). Let $X_1, X_2,\ldots, X_k$ and $Y_1, Y_2, \ldots, Y_k$ be two sets of independent random variables. If $X_j \le _{\mathrm{cx}} Y_{j}$ for $j=1,2,\ldots,k$, then
  12 $$\begin{aligned} \sum _{j=1}^{k} X_{j} \le _{\mathrm{cx}} \sum _{j=1}^{k} Y_{j}. \end{aligned}$$
- Combining the properties of closure under mixtures and closure under convolutions, we see the convex order is *closed under random sums* so
  13 $$\begin{aligned} \sum _{j=1}^{K} X_{j} \le _{\mathrm{cx}} \sum _{j=1}^{K} Y_{j}, \end{aligned}$$
  for any non-negative integer random variable *K*. As an application of the closure under random sums property of the convex order, consider two random variables *K* and $\tilde{K}$ that related by binomial thinning. That is, $G_{\tilde{K}}(z) = G_{K}(1 - p + pz)$ for some $p \in (0,1)$. Then $$K \le _{\mathrm{Lorenz}} \tilde{K}\ $$ (McVinish and Lester 2020, Section 3)
- The closure under random sums property can be adapted to the case where the $X_1, X_2, \ldots$ and $Y_1, Y_2, \ldots$ are two iid sequences with $X \le _{\mathrm{cx}} Y$, and $K_1$ and $K_2$ are non-negative integer random variables such that $K_{1} \le _{\mathrm{cx}} K_{2}$. In this case, (Shaked and Shanthikumar 2007, Theorem 3.A.13) implies
  14 $$\begin{aligned} \sum _{j=1}^{K_1} X_{j} \le _{\mathrm{cx}} \sum _{j=1}^{K_2} Y_{j}. \end{aligned}$$
- The survival function can be used to establish if two random variables can be compared in the convex order. If *X* and *Y* are two random variables with the same mean and $\bar{F}_X - \bar{F}_Y$ has a single sign change from positive to negative, then $$X \le _{\mathrm{cx}} Y\ $$ (Shaked and Shanthikumar 2007, Theorem 3.A.44(b)). This property can also be used to characterise the convex order (Shaked and Shanthikumar 2007, Theorem 3.A.45).

### Measures of aggregation

In practice, levels of aggregation are compared with numerical summaries rather than using the entire Lorenz curve. If we accept the Lorenz order as the way to compare aggregation in parasite-host systems (Poulin 1993; McVinish and Lester 2020), then our measures of aggregation should respect the Lorenz order. That is, if $X \le _{\mathrm{Lorenz}} Y$, then the measure of aggregation $I(\cdot )$ should satisfy $I(X) \le I(Y)$. Arnold and Sarabia (2018, Chapter 5) review several inequality measures and these can be applied as measures of aggregation. We restrict our attention in this paper to the following four measures respecting the Lorenz order; the coefficient of variation, the Gini index, the Pietra index (also known as the Hoover index, or the Robin-Hood index) and $1 - {\text {prevalence}}$.

The **coefficient of variation** is given by

15 $$\begin{aligned} CV(X) = \frac{\sqrt{\text {Var}(X)}}{\mathbb {E} \left[ X\right] }. \end{aligned}$$

This measure is rarely used in parasitology, though it is mentioned in some reviews on parasite aggregation such as Wilson et al. (2001) and McVinish and Lester (2020). As means and variances are commonly reported in empirical studies and are often easily calculated for theoretical models, it may be useful in some contexts. For example, from Eqs. (2) and (3), the squared coefficient of variation for the Tallis-Leyton model is

16 $$\begin{aligned} \text {CV}^{2}(M(a)) = \frac{1}{\lambda } \frac{\mathbb {E} \left[ N(N-1)\right] }{(\mathbb {E} \left[ N\right] )^{2}} \frac{\int ^a_0 \bar{F}^{2}_{T}(s) ds}{ \left( \int ^a_0 \bar{F}_{T}(s) ds \right) ^{2}} + \frac{1}{\lambda \mathbb {E} \left[ N\right] \int ^a_0 \bar{F}_{T}(s) ds}. \end{aligned}$$

The **Gini index** (Gini 1914, 2005) is given by twice the area between the egalitarian line and the Lorenz curve. For a random variable *X*, the Gini index can be expressed as

17 $$\begin{aligned} G(X) = \frac{\mathbb {E}\,\left[|X - X'|\right] }{2\, \mathbb {E} \left[ X\right] }, \end{aligned}$$

where $X'$ is an independent random variable with $$X'{\mathop {=}\limits ^{d}} X\ $$ (Arnold and Sarabia 2018, Page 47). The **Pietra index** is given by the maximum vertical distance between the egalitarian line and the Lorenz curve (Pietra 1915, 2014). McVinish and Lester (2020) argue that this index could be useful due to its simple interpretation as the proportion of the parasite population that would need to be redistributed among the hosts in order for all hosts to have the same parasite load. The Pietra index can be expressed as

18 $$\begin{aligned} P(X) = \frac{\mathbb {E}\left[ \left| X - \mathbb {E} \left[ X\right] \right| \right] }{2\, \mathbb {E} \left[ X\right] }, \end{aligned}$$

(Arnold and Sarabia 2018, Lemma 5.3.1). In general, the dependence of the Pietra index on the mean is not smooth. For example, the Pietra index for the Poisson distribution with mean $\mu >0$ is

19 $$\begin{aligned} \frac{e^{-\mu } \mu ^{m-1}}{(m-1)!}, \end{aligned}$$

where *m* is the smallest integer greater than or equal to $$\mu\ $$ (Ramasubban 1958). While the Pietra index in this instance is continuous in $\mu$, it is not differentiable with respect to $\mu$ at integer values of $\mu$. Similar behaviour will be observed in the numerical results reported in Sect. 3.

**Prevalence**, the probability that a host is infected by at least one parasite, is an important quantity in parasitology (Jovani and Tella 2006; Kura et al. 2022). Although prevalence is not usually thought of as a measure of aggregation, we may express $$ 1 - {\text {prevalence}} $$ in terms of the Lorenz curve. From the definition of the Lorenz curve, $$L(u) = 0$$ if $$F^{-1}(u) = 0$$. From the definition of the quantile function, $$ F^{-1}(u) = 0 $$ for $$ u < F(0)$$. As the Lorenz curve is continuous and $$F(0) = 1 - {\text {prevalence}}$$, we see

20 $$\begin{aligned} 1 - {\text {prevalence}} = \max \{u: L(u) = 0 \}. \end{aligned}$$

Prevalence for the Tallis-Leyton model can be evaluated directly from the PGF as

21 $$\begin{aligned} 1 - {\text {prevalence}} = G_{M}(0;a) = \exp \left( \lambda \int _{0}^{a} \left[ G_{N}(1 - \bar{F}_{T}(a-s)) -1 \right] ds\right). \end{aligned}$$

There is a close connection between the Pietra index and prevalence. If $\mathbb {E}\left[ X\right] \le 1$, then

22 $$\begin{aligned} \mathbb {E}\left[|X-\mathbb {E}[X]|\right]&= \mathbb {E}[X] \mathbb {P}(X=0) + \sum _{k=1}^{\infty } \left( k-\mathbb {E}[X]\right) \mathbb {P}(X=k) \end{aligned}$$

23 $$\begin{aligned}&= 2 \mathbb {E}[X] \mathbb {P}(X=0). \end{aligned}$$

Hence,

24 $$\begin{aligned} \text {if } \mathbb {E}\left[ X\right] \le 1, \text { then } P(X) = 1 - {\text {prevalence}}. \end{aligned}$$

More generally, the four indices are constrained by the following inequality

25 $$\begin{aligned} 1 - {\text {prevalence}} \le P(X) \le G(X) \le P(X) (2- P(X)) \le CV(X) \end{aligned}$$

(Taguchi 1968; McVinish and Lester 2020).

The Gini index and Pietra index can be further related to the coefficient of variation when the distribution of parasites is approximately normal. Suppose $X_{1}, X_{2}, \ldots$ is a sequence of random variables such that

26 $$\begin{aligned} \frac{X_{n} - \mathbb {E}\left[ X_{n}\right] }{\sqrt{\text {Var}(X_{n})}} =: Z_{n} {\mathop {\rightarrow }\limits ^{d}} Z, \end{aligned}$$

where $Z \sim {\mathsf{N}}(0,1)$. As $X_{n} \ge 0$ with probability one, the above limit is only possible if $CV(X_n) \rightarrow 0$. Nevertheless, the ratio of the Gini index to the coefficient of variation still has a well defined limit. The Gini index of $X_n$ can be expressed as

27 $$\begin{aligned} G(X_{n}) = \frac{\mathbb {E} \left[|X_{n} - X_{n}'|\right] }{2 \mathbb {E} \left[ X_{n}\right] } = \frac{\sqrt{\text {Var}(X_{n})}}{2\mathbb {E} \left[ X_{n}\right] } \mathbb {E}\left[|Z_{n} - Z_{n}'|\right], \end{aligned}$$

where $Z_{n}'$ is an independent random variable with $Z_{n}' {\mathop {=}\limits ^{d}} Z_{n}$. Since $\mathbb {E} \left[ Z_{n}^{2}\right] = 1$, the collection of random variables $\{Z_{n} - Z_{n}'\}$ is uniformly integrable and $\mathbb {E} \left[|Z_{n} - Z_{n}'|\right] \rightarrow \mathbb {E}\left[|Z - Z'|\right] = 2/\sqrt{\pi }$. Applying the asymptotic normality and uniform integrability of the $Z_{n}$,

28 $$\begin{aligned} \frac{G(X_{n})}{CV(X_{n})} \rightarrow \frac{1}{\sqrt{\pi }}. \end{aligned}$$

Similarly, the Pietra index of $X_n$ can be expressed as

29 $$\begin{aligned} P(X_{n}) = \frac{\mathbb {E} \left[ \left| X_{n} - \mathbb {E} \left[ X_{n}\right] \right| \right] }{2 \mathbb {E} \left[ X_{n}\right] } = \frac{\sqrt{\text {Var}(X_{n})}}{2\mathbb {E} \left[ X_{n}\right] } \mathbb {E}\left[|Z_{n}|\right]. \end{aligned}$$

Applying the asymptotic normality and uniform integrability of the $Z_{n}$,

30 $$\begin{aligned} \frac{P(X_{n})}{CV(X_{n})} \rightarrow \frac{1}{\sqrt{2\pi }}. \end{aligned}$$

### Numerical evaluation of aggregation measures from the PGF

From Eq. (16), the coefficient of variation can be relatively easily evaluated for the Tallis-Leyton model. Numerical integration of $\bar{F}_{T}(s)$ and $\bar{F}_{T}^2(s)$ may be required, but the dependence on age and $\lambda$ is explicit. Similarly, $1 - {\text {prevalence}}$ could be evaluated with a single numerical integration using (21). On the other hand, evaluation of the Gini and Pietra indices require evaluation of the probability mass function. In the examples of the next section, we numerically evaluate the probability mass function of *M*(*a*) by inverting $G_{M}(z;a)$ using the Abate-Whitte algorithm (Abate and Whitt 1992). The algorithm was implemented in MATLAB (The MathWorks Inc. 2022a) using the vpa function in the Symbolic Math Toolbox (The MathWorks Inc. 2022b) for high precision arithmetic. The code used to evaluate the indices is available from McVinish (2025).

## Analysis of the Tallis-Leyton model

In this section we characterize how the different processes in the Tallis-Leyton model shape parasite aggregation in the sense of the Lorenz ordering and the related indices discussed in Sect. 2.3. The analysis begins with a representation of the host’s parasite load, *M*(*a*), as a random variable having a compound Poisson distribution. This representation is used extensively to understand how the rate of infectious contacts ($\lambda$), the distribution of the number of parasites (*N*) that enter the host during an infectious contact, the age of the host (*a*), and lifetime distribution of the parasites (*T*) all affect the distribution of a host’s parasite load in terms of the Lorenz order. When comparing the host’s parasite load in two systems, the parameters of the second parasite-host system is distinguished by a tilde.

### Compound Poisson representation

Let *n* be a non-negative integer, $v \in [0,1]$, and let *X*(*n*, *v*) denote a random variable from a ${\mathsf{Bin}}(n,v)$ distribution, with $X(n,v) = 0$ with probability 1 when $n=0$. Our first result will represent a host’s parasite load *M*(*a*) as a random sum of independent and identically distributed random variables.

#### Theorem 1

Assume *N* has a distribution on the non-negative integers and *T* has a continuous distribution on $[0,\infty )$. For $a > 0$, define *V* to be a random variable on $[\bar{F}_{T}(a),1]$ with distribution function

31 $$\begin{aligned} F_{V}(v) = 1 - a^{-1} \bar{F}^{-1}_{T}(v). \end{aligned}$$

Let $X_{1},X_{2},\ldots$ be a sequence of independent random variables with the same distribution as *X*(*N*, *V*), where *N* and *V* are independent. Let $\Lambda (t)$ be a Poisson process with rate $\lambda$ that is independent of the sequence $X_1, X_2, \ldots$ Then

32 $$\begin{aligned} M(a) {\mathop {=}\limits ^{d}} \sum _{k=1}^{\Lambda (a)} X_{k}. \end{aligned}$$

#### Proof

We first determine the PGF of *X*(*N*, *V*). The PGF of *X*(*n*, *v*) is $G_{X(n,v)}(z) = (1 + v(z-1))^{n}$. By conditioning on *N*, the PGF of *X*(*N*, *v*) is seen to be

33 $$\begin{aligned} G_{X(N,v)}(z) = \mathbb {E} \left[ (1+ v(z-1))^{N} \right] = G_{N}\left( 1 + v (z-1) \right). \end{aligned}$$

By conditioning on *V* and then applying the distribution function of *V* (31), we can write the PGF of *X*(*N*, *V*) as

34 $$\begin{aligned} G_{X(N,V)}(z)&= \mathbb {E} \left[ G_{N}\left( 1+ V(z-1)\right) \right] \end{aligned}$$

35 $$\begin{aligned}&= \int _{\bar{F}_{T}(a)}^{1} G_{N}\left( 1 + v(z-1) \right) \, d \left[ 1 - a^{-1} \bar{F}^{-1}_{T}(v)\right]. \end{aligned}$$

We now determine the PGF of the right-hand side of Eq. (32). Conditioning on $\Lambda (a)$ and noting that $X_{1},X_{2},\ldots$ is a sequence of independent random variables with the same distribution as *X*(*N*, *V*), the PGF of $\sum _{k=1}^{\Lambda (a)} X_{k}$ is seen to be

36 $$\begin{aligned} G_{ \sum _{k=1}^{\Lambda (a)} X_{k}}(z)&= \mathbb {E} \left[ G_{X(N,V)}(z)^{\Lambda (a)} \right] \end{aligned}$$

37 $$\begin{aligned}&= \exp \left\{ \lambda a \left( \int _{\bar{F}_{T}(a)}^{1} G_{N}\left( 1 + v(z-1) \right) d \left[ 1 - a^{-1} \bar{F}^{-1}_{T}(v)\right] -1 \right) \right\} \end{aligned}$$

38 $$\begin{aligned}&= \exp \left\{ \lambda \int _{\bar{F}_{T}(a)}^{1} \left[ G_{N}\left( 1 + v(z-1) \right) - 1 \right] d \left[ a - \bar{F}^{-1}_{T}(v)\right] \right\}. \end{aligned}$$

Upon making the substitution $v = \bar{F}_{T}(a-s)$ so $s = a - \bar{F}_{T}^{-1}(v)$, the PGF of $\sum _{k=1}^{\Lambda (a)} X_{k}$ can be expressed as

39 $$\begin{aligned} G_{\sum _{k=1}^{\Lambda (a)} X_{k}}(z)&= \exp \left( \lambda \int _{0}^{a} \left[ G_{N}(1 + \bar{F}_{T}(a-s) (z-1)) -1 \right] ds\right), \end{aligned}$$

which by Eq. (1) is $G_{M}(z;a)$. $\square$

### Rate of infectious contacts

In this section we examine the effect of the rate of infectious contacts ($\lambda$) on the parasite aggregation. The rate of infectious contacts has no effect on the variance-to-mean ratio (4), whereas the coefficient of variation is strictly decreasing as the rate of infectious contacts increases (16). The following result shows an increase in the rate of infectious contacts decreases parasite aggregation in the sense of the Lorenz order.

#### Theorem 2

If $\tilde{\lambda } < \lambda$ and all other model parameters are equal, then $M(a) \le _{\mathrm{Lorenz}} \tilde{M}(a)$.

#### Proof

Set $$\kappa = \tilde{\lambda }/\lambda $$. Let $$ X_{1}, X_{2}, \ldots $$ be a sequence of independent random variables having the same distribution as *X*(*N*, *V*) and let $$B_{1},B_{2},\ldots $$ be a sequence of independent $${\mathsf{Ber}}(\kappa )$$ random variables that are also independent of the $$X_{k}$$. As $$ \kappa \le _{\mathrm{cx}} B_{k} $$ and the convex order is closed under mixtures, $$ \kappa X_{k} \le _{\mathrm{cx}} B_{k} X_{k}$$. The PGF of $$ B_{k} X_{k}$$ is $$G_{ B_{k} X_{k}}(z) = \kappa \, G_{X(N,V)}(z) + 1-\kappa $$. Let $$\Lambda (t)$$ be a Poisson process with rate $$\lambda $$. As the convex order is closed under random sums,

40 $$\begin{aligned} \kappa \sum _{k=1}^{\Lambda (a)} X_{k} \le _{\mathrm{cx}} \sum _{k=1}^{\Lambda (a)} B_{k} X_{k}. \end{aligned}$$

By Theorem 1, $\sum _{k=1}^{\Lambda (a)} X_{k} {\mathop {=}\limits ^{d}} M(a)$. To determine the distribution of $\sum _{k=1}^{\Lambda (a)} B_{k} X_{k}$, we evaluate its PGF

41 $$\begin{aligned} G_{\sum _{k=1}^{\Lambda (a)} B_{k} X_{k}}(z)&= \exp \left\{ \lambda a\left[ (\kappa \, G_{X(N,V)}(z) + 1-\kappa ) -1 \right] \right\} \end{aligned}$$

42 $$\begin{aligned}&= \exp \left\{ \tilde{\lambda } a \left[ G_{X(N,V)}(z) -1 \right] \right\} = G_{\tilde{M}}(a;z). \end{aligned}$$

Hence, $$ M(a) \le _{\mathrm{Lorenz}} \tilde{M}(a)$$. $$\square $$

**Fig. 1:**
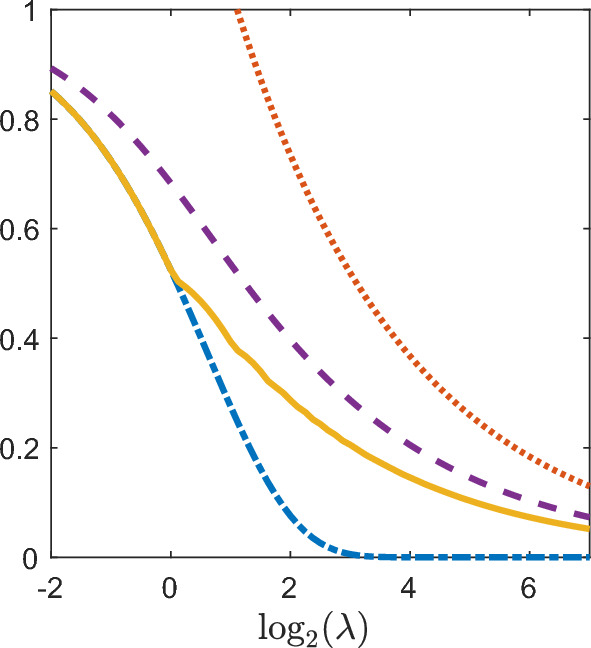
Plot of the coefficient of variation (orange dotted line), Gini index (purple dashed line), Pietra index (yellow solid line), and $$ 1 - {\text {prevalence}}\ $$ (blue dot-dashed line) for a host aged 3 in the Tallis-Leyton model with $N \sim {\mathsf{NB}}(1,1)$, and $T \sim {\mathsf{Exp}}(1)$. Since $\mathbb {E}\left[ M(3)\right] \le 1$ for $\lambda \le 1.05$, the Pietra index and $1 - {\text {prevalence}}$ coincide on that interval of $\lambda$ as expected (24)

Figure 1 shows the four indices (coefficient of variation, Gini, Pietra, and $1 - {\text {prevalence}}$) for a host aged 3 with rate of infectious contacts ($\lambda$) in [0.25, 128], the number of parasites (*N*) entering the host at infectious contacts having a ${\mathsf{NB}}(1,1)$ distribution, and the parasite lifetimes (*T*) having a ${\mathsf{Exp}}(1)$ distribution. All four indices are strictly decreasing as the rate of infectious contacts increases. The coefficient of variation (16) is not displayed for small values of $\lambda$ as it is proportional to $\lambda ^{-1/2}$. For $\lambda \le 1.05$, the expected parasite load is less than one so the Pietra index and $1 - {\text {prevalence}}$ are equal for $\lambda \le 1.05$ following (24). As expected from the discussion in Sect. 2.3, the Pietra index appears to display some discontinuity in the first derivative at points where the expected parasite load is integer valued. This behaviour is less apparent at larger values of $\lambda$.

### Distribution of *N*

We now consider the role of the distribution of the number of parasites (*N*) that enter the host during an infectious contact. As a concrete example, suppose $$N \sim {\mathsf{NB}}(m,k)$$, where *m* is the mean and the variance is $$ m + m^{2}/k$$. From (4), the variance-to-mean ratio of the parasite load *M*(*a*) is

43 $$\begin{aligned} \text {VMR}(M(a)) = 1 + m \left( 1+\frac{1}{k}\right) \frac{\int ^a_0 \bar{F}^{2}_{T}(s) ds}{\int ^a_0 \bar{F}_{T}(s) ds}. \end{aligned}$$

We see that the variance-to-mean ratio is increasing in *m* but decreasing in *k*. In contrast, the coefficient of variation of *M*(*a*) is decreasing in both *m* and *k*.

The next two results show increased variability in the number of parasites entering the host during an infectious contact leads to increased parasite aggregation in the sense of the Lorenz order. The first of these results uses the convex order, which requires the distributions being compared to have the same expectation.

#### Theorem 3

Suppose that *N* and $\tilde{N}$ are non-negative integer valued random variables such that $\mathbb {E}\left[ N\right] = \mathbb {E}[\tilde{N}]$ and $N \le _{\mathrm{cx}} \tilde{N}$. Assume that all other model parameters are equal. Then $M(a) \le _{\mathrm{cx}} \tilde{M}(a)$.

#### Proof

Using an extension of the closure under random sums property of the convex order Shaked and Shanthikumar (2007, Theorem 3.A.13),

44 $$\begin{aligned} X(N,v) \le _{\mathrm{cx}} X(\tilde{N},v). \end{aligned}$$

As the convex order is closed under mixtures, $X(N,V) \le _{\mathrm{cx}} X(\tilde{N},V)$. Let $X_{1}, X_{2}, \ldots$ be a sequence of independent random variables having the same distribution as *X*(*N*, *V*) and let $\tilde{X}_{1}, \tilde{X}_{2},\ldots$ be a sequence of independent random variables having the same distribution as $X(\tilde{N},V)$. As the convex order is closed under random sums,

45 $$\begin{aligned} \sum _{k=1}^{\Lambda (a)} X_{k} \le _{\mathrm{cx}} \sum _{k=1}^{\Lambda (a)} \tilde{X}_{k}. \end{aligned}$$

Theorem 1 shows $$ M(a) \le _{\mathrm{cx}} \tilde{M}(a)$$. $$\square $$

For distributions with different means, we consider only the case where *N* and $\tilde{N}$ are related by binomial thinning. Recall that if $G_{\tilde{N}}(z) = G_{N}(1-p+pz)$ for some $p \in (0,1)$, then $\tilde{N} \le _{\mathrm{Lorenz}} N$.

#### Theorem 4

Suppose that $G_{\tilde{N}}(z) = G_{N}(1 - p + pz)$ for some $p \in (0,1)$ and all other model parameters are equal. Then $M(a) \le _{\mathrm{Lorenz}} \tilde{M}(a)$.

#### Proof

Let $U_1, U_2, \dots$ and $U'_1, U'_2, \ldots$ be independent standard uniform random variables. Then standard conditioning arguments show

46 $$\begin{aligned} X(\tilde{N},v) {\mathop {=}\limits ^{d}} \sum _{j=1}^{N} \mathbb {I}\left( U_{j} \le v \right) \mathbb {I}\left( U'_{j} \le p \right). \end{aligned}$$

As the convex order is closed under mixtures,

47 $$\begin{aligned} p\, \mathbb {I}\left( U_{j} \le v \right) \le _{\mathrm{cx}} \mathbb {I}\left( U_{j} \le v \right) \mathbb {I}\left( U'_{j} \le p \right). \end{aligned}$$

As the convex order is closed under random sums, $p X(N,v) \le _{\mathrm{cx}} X(\tilde{N},v)$. Following the same arguments as in the proof of Theorem 3, we see $p M(a) \le _{\mathrm{cx}} \tilde{M}(a)$. Hence, $M(a) \le _{\mathrm{Lorenz}} \tilde{M}(a)$. $\square$

When the distribution of the number of parasite has a ${\mathsf{NB}}(m,k)$ distribution, Theorems 3 and 4 together show that an increase in *m* or *k* will decrease parasite aggregation in the sense of the Lorenz order.

#### Corollary 5

Suppose $N \sim {\mathsf{NB}}(m, k)$ and $\tilde{N} \sim {\mathsf{NB}}(\tilde{m}, \tilde{k})$ with $\tilde{k} \le k$ and $\tilde{m} \le m$. Assume that all other model parameters are equal. Then $M(a) \le _{\mathrm{Lorenz}} \tilde{M}(a)$.

#### Proof

Let $\hat{M}(a)$ be the parasite load for a host of age *a* in the Tallis-Leyton model with $\hat{N} \sim {\mathsf{NB}}(\tilde{m}, k)$ and all other model parameters equal. The PGF of the ${\mathsf{NB}}(m, k)$ distribution is

48 $$\begin{aligned} G_{N}(z) = \left( \frac{k}{k + m - mz} \right) ^{k} \end{aligned}$$

and $G_{\hat{N}}(z) = G_{N}(1 - p + pz)$ with $p = \tilde{m}/m$. Theorem 4 implies $M(a) \le _{\mathrm{Lorenz}} \hat{M}(a)$. Since $\mathbb {E}[\hat{N}] = \mathbb {E}[\tilde{N}]$ and ${\mathsf{NB}}(\tilde{m}, k) \le _{\mathrm{Lorenz}} {\mathsf{NB}}(\tilde{m}, \tilde{k})$, $\hat{N} \le _{\mathrm{cx}} \tilde{N}$. Theorem 3 implies $\hat{M}(a) \le _{\mathrm{cx}} \tilde{M}(a)$. As the Lorenz ordering is transitive, $M(a) \le _{\mathrm{Lorenz}} \tilde{M}(a)$. $\square$

**Fig. 2:**
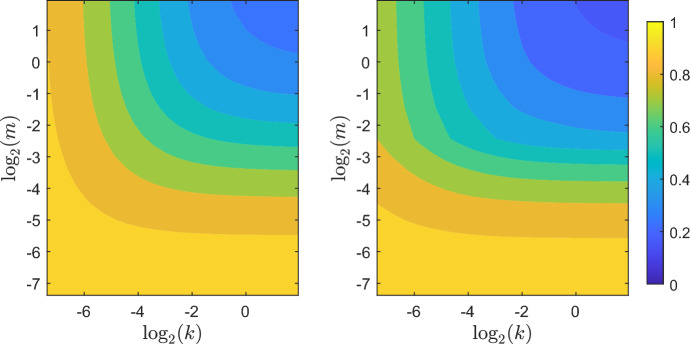
Contour plots showing Gini index (Left) and Pietra index (Right) for a host aged 10 in the Tallis-Leyton model with $\lambda = 5$, $N \sim {\mathsf{NB}}(m,k)$ and $T \sim {\mathsf{Exp}}(1)$

Figure 2 shows the Gini and Pietra indices for a parasite host system with host aged 10, rate of infectious contacts $\lambda = 5$, the distribution of the number of parasites (*N*) that enter the host during an infectious contact following a ${\mathsf{NB}}(m,k)$ distribution, and parasite lifetimes (*T*) having an ${\mathsf{Exp}}(1)$ distribution. Both indices are decreasing in both *m* and *k* as we expect from the above results. The contours of both the Gini and Pietra indices tend to become parallel to the respective axes as $m \rightarrow \infty$ and $k \rightarrow \infty$. This is a consequence of the limiting behaviour of the negative binomial distribution (Adell and Cal 1994). The contours of the Pietra index display some discontinuity in the first derivative for $m = 1/5 (\log _2(m) \approx -2.3)$, which corresponds to a host’s expected parasite load being 1.

It is natural to consider which distribution for *N* results in the least aggregated distribution for the host’s parasite load. This requires determining the smallest distribution in the convex ordering. The distributions being compared must have the same expected value. Let *n* be a non-negative real number. Define the random variable *N* such that

49 $$\begin{aligned} \mathbb {P}(N = \lfloor n \rfloor )&= 1 - (n - \lfloor n \rfloor )&\mathbb {P}(N = \lfloor n \rfloor +1)&= n - \lfloor n \rfloor. \end{aligned}$$

In the supplementary material of McVinish and Lester (2020) it was shown for any random variable $\tilde{N}$ on the non-negative integers with $\mathbb {E}[\tilde{N}] = n$ is larger than *N* in the convex order. That is, $N \le _{\mathrm{cx}} \tilde{N}$ and we can say *N* has the smallest distribution in convex order with expectation *n*. When $n \le 1$, the smallest distribution in convex order for *N* leads to *M*(*a*) having a Poisson distribution. There is no largest distribution in the convex order.

### Host age

We now examine the effect of the host’s age (*a*) on parasite aggregation. Differentiating (4) with respect to *a* shows the variance-to-mean ratio is a decreasing function of the host’s age. Since the expected parasite load is increasing in age, the coefficient of variation is also decreasing in the host’s age. The following result shows that parasite aggregation in the sense of Lorenz order decreases as the host age increases.

#### Theorem 6

If $$ \tilde{a} <a $$, then $$ M(a) \le _{\mathrm{Lorenz}} M(\tilde{a})$$.

The proof is built from the following lemmas.

#### Lemma 7

Let *V* have the distribution (31) and let $\tilde{V}$ have the distribution (31) with *a* replaced by $\tilde{a}$. Let $B \sim {\mathsf{Ber}}(\mu (\tilde{a})/\mu (a))$ independent of *V*, and let $\tilde{B} \sim {\mathsf{Ber}}(\tilde{a}/a)$ independent of $\tilde{V}$. Then $BV \le _{\mathrm{cx}} \tilde{B} \tilde{V}$.    

#### Proof

Note that

50 $$\begin{aligned} \mathbb {E} \left[ V\right] = \frac{1}{a} \int _{0}^{a} \bar{F}_{T}(s) ds \end{aligned}$$

so $\mathbb {E} \left[ BV\right] = \mathbb {E} [\tilde{B}\tilde{V}]$. We show that $BV \le _{\mathrm{cx}} \tilde{B} \tilde{V}$ by examining the sign changes of $\bar{F}_{BV} - \bar{F}_{\tilde{B}\tilde{V}}$. The survival functions of *BV* and $\tilde{B}\tilde{V}$ are

51 $$\begin{aligned} \bar{F}_{BV}(w)&= \left\{ \begin{array}{ll} \frac{\mu (\tilde{a})}{\mu (a)}, & w \in [0,\bar{F}_{T}(a)) \\ \frac{\mu (\tilde{a})}{a\, \mu (a)} \bar{F}^{-1}_{T}(w), & w \in [\bar{F}_{T}(a),1) \\ 0, & w > 1, \end{array} \right. \end{aligned}$$

and

52 $$\begin{aligned} \bar{F}_{\tilde{B}\tilde{V}}(w)&= \left\{ \begin{array}{ll} \frac{\tilde{a}}{a}, & w \in [0,\bar{F}_{T}(\tilde{a})) \\ \frac{1}{a} \bar{F}^{-1}_{T}(w), & w \in [\bar{F}_{T}(\tilde{a}),1) \\ 0, & w > 1. \end{array} \right. \end{aligned}$$

Since $\mu (a)$ is increasing in *a* and $\mu (a)/a$ is decreasing in *a*,

53 $$\begin{aligned} \frac{\tilde{a}}{a}< \frac{\mu (\tilde{a})}{\mu (a)} < 1. \end{aligned}$$

Hence, $\bar{F}_{BV}(w) - \bar{F}_{\tilde{B}\tilde{V}}(w) > 0$ for all $w \in [0, \bar{F}_{T}(a)]$. On $[\bar{F}_{T}(a), \bar{F}_{T}(\tilde{a})]$, $\bar{F}_{\tilde{B}\tilde{V}}(w) = \tilde{a}/a$ whereas $\bar{F}_{BV}$ decreases from $\mu (\tilde{a})/\mu (a)$ to $\tilde{a} \mu (\tilde{a}) /a \mu (a) < \tilde{a}/a$. For all $w \ge \bar{F}_{T}(\tilde{a})$, $\bar{F}_{BV}(w) - \bar{F}_{\tilde{B}\tilde{V}}(w) < 0$. Hence, $\bar{F}_{BV} - \bar{F}_{\tilde{B}\tilde{V}}$ has a single sign change from positive to negative. Hence, $BV \le _{\mathrm{cx}} \tilde{B}\tilde{V}$ (Shaked and Shanthikumar 2007, Theorem 3.A.44). $\square$

#### Lemma 8

For any convex function $\phi$ and any non-negative integer valued random variable *N* that is independent of $U_{1},U_{2},\ldots$, $\mathbb {E}\left[ \phi (X(N,v))\right]$ is a convex function in *v*.

#### Proof

As the binomial distribution ${\mathsf{Bin}}(n,v)$ is a regular exponential family of distribution with expectation linear in *v*, Schweder (1982, Proposition 2) implies $\mathbb {E} \left[ \phi (X(n,v)\right]$ is convex in *v* for any positive integer *n*. As non-negative weighted sums of convex functions are also convex, it follows that $\mathbb {E} \left[ \phi (X(N,v)) \right]$ is a convex function in *v*. $\square$

#### Proof of Theorem 6

As $X(n,v) \sim {\mathsf{Bin}}(n,v)$, if *b* takes values in $\{0,1\}$, then $b X(n,v) = X(n,bv)$. Applying Shaked and Shanthikumar (2007, Theorem 3.A.21) with Lemmas 7 and 8,

54 $$\begin{aligned} BX(N,V) = X(N,BV) \le _{\mathrm{cx}} X(N, \tilde{B} \tilde{V}) = \tilde{B} X(N, \tilde{V}). \end{aligned}$$

Since the convex order is transitive and closed under mixtures,

55 $$\begin{aligned} \frac{\mu (\tilde{a})}{\mu (a)} X(N,V) \le _{\mathrm{cx}} B X(N,V) \le _{\mathrm{cx}} \tilde{B} X(N, \tilde{V}). \end{aligned}$$

In the notation of Theorem 1, $M(a) {\mathop {=}\limits ^{d}} \sum _{k=1}^{\Lambda (a)} X_{k}$, where $X_1,X_2,\ldots$ is a sequence of independent random variables with $X_k {\mathop {=}\limits ^{d}} X(N,V)$. From the thinning property of the Poisson process and Theorem 1, we can write $M(\tilde{a}) {\mathop {=}\limits ^{d}} \sum _{k=1}^{\Lambda (a)} \tilde{B}_{k} \tilde{X}_{k}$, where $\tilde{X}_{1},\tilde{X}_{2},\ldots$ is a sequence of independent random variables with $\tilde{X}_{k}{\mathop {=}\limits ^{d}} X(N,\tilde{V})$ and $\tilde{B}_{1},\tilde{B}_{2},\ldots$ is a sequence of independent ${\mathsf{Ber}}(\tilde{a}/a)$ random variables that are also independent of the $\tilde{X}_{k}$. As the convex order is closed under random sums,

56 $$\begin{aligned} \frac{\mu (\tilde{a})}{\mu (a)} M(a) {\mathop {=}\limits ^{d}} \frac{\mu (\tilde{a})}{\mu (a)} \sum _{k=1}^{\Lambda (a)} X_{k} \le _{\mathrm{cx}} \sum _{k=1}^{\Lambda (a)} B_{k} X_{k} \le _{\mathrm{cx}} \sum _{k=1}^{\Lambda (a)} \tilde{B}_{k} \tilde{X}_{k} {\mathop {=}\limits ^{d}} M(\tilde{a}) \end{aligned}$$

$$\square $$    

**Fig. 3:**
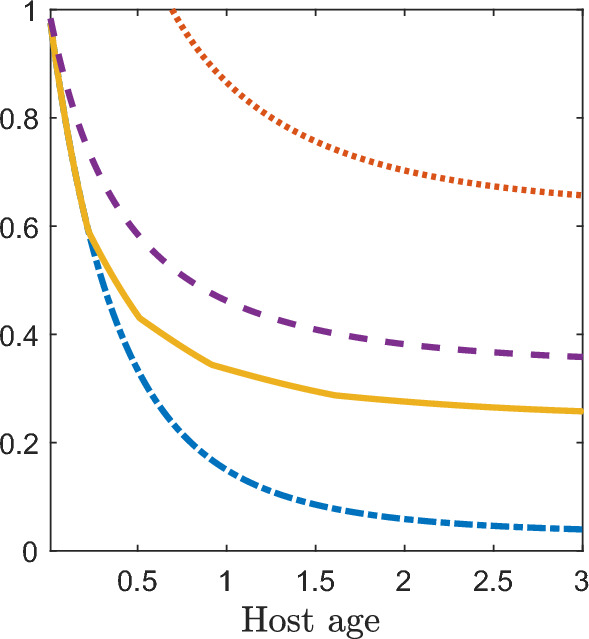
Plot of the coefficient of variation (orange dotted line), Gini index (purple dashed line), Pietra index (yellow solid line), and $$ 1 - {\text {prevalence}}\ $$ (blue dot-dashed line) for a host in the Tallis-Leyton model with $\lambda = 5$, $N \sim {\mathsf{NB}}(1,1)$, and $T \sim {\mathsf{Exp}}(1)$. Since $\mathbb {E}\left[ M(a)\right] \le 1$ for $a\le -\ln (4/5) \approx 0.22$, the Pietra index and $1 - {\text {prevalence}}$ coincide for $a \le 0.22$ as expected (24)

Figure 3 shows the four indices (coefficient of variation, Gini, Pietra, and $1 - {\text {prevalence}}$) for the parasite load *M*(*a*) of a host aged *a* in the Tallis-Leyton model with rate of infectious contacts $\lambda = 5$, the number of parasites (*N*) entering the host during an infectious contact following a ${\mathsf{NB}}(1,1)$ distribution, and parasite lifetimes (*T*) following an ${\mathsf{Exp}}(1)$ distribution. All four indices are strictly decreasing in host age. The Pietra index appears to be crudely interpolated, however all indices were evaluated on the same grid with a step size of 0.01. The ages where the Pietra index appears non-differentiable are those ages where the expected parasite load of the host is integer valued. Specifically, the expected parasite load of the host is $\mathbb {E}\left[ M(a)\right] = 5(1-\exp (-a))$ so the host has integer valued expected parasite load at ages 0.22, 0.51, 0.92 and 1.61. As in Fig. 1, the Pietra index coincides with $1 - {\text {prevalence}}$ for $\mathbb {E}\left[ M(a)\right] \le 1$, that is for $a \le 0.22$.

### Parasite lifetime distribution

We now assess the effect of variability in the parasite lifetime distribution (*T*) on parasite aggregation. Rather than assuming $T \le _{\mathrm{cx}} \tilde{T}$, we will assume that $\mathbb {E} \left[ T\right] = \mathbb {E} [\tilde{T}]$ and $\bar{F}_{T} - \bar{F}_{\tilde{T}}$ has a single sign change from positive to negative. As noted in the last bullet point of Sect. 2.2, these conditions imply $T \le _{\mathrm{cx}} \tilde{T}$. The below result shows that increased variability in the parasite lifetimes decreases parasite aggregation in the sense of the Lorenz order. In particular, the result implies that the host’s parasite load is most aggregated when parasites have constant lifetimes.

#### Theorem 9

Suppose $\mathbb {E} \left[ T\right] = \mathbb {E} [\tilde{T}]$ and $\bar{F}_{T} - \bar{F}_{\tilde{T}}$ has a single sign change from positive to negative. Assume all other model parameters are equal. Then $\tilde{M}(\infty ) \le _{\mathrm{cx}} M(\infty )$.

#### Proof

We first show that for all $$ a> 0$$,

57 $$\begin{aligned} \int _{0}^{a} \bar{F}_{T}(t) dt \ge \int _{0}^{a} \bar{F}_{\tilde{T}}(t) dt. \end{aligned}$$

Define the function $H:[0,\infty ) \rightarrow \mathbb {R}$ as

58 $$\begin{aligned} H(a) = \int _{0}^{a} \left( \bar{F}_{T}(t) - \bar{F}_{\tilde{T}}(t) \right) dt. \end{aligned}$$

By definition $$H(0) = 0$$. As $$\bar{F}_{T} - \bar{F}_{\tilde{T}}$$ has a single sign change from positive to negative, *H* first increases and then decreases on $$[0,\infty )$$. Since $$ \mathbb {E} \left[ T\right] = \mathbb {E} [\tilde{T}]$$, $$\lim _{a\rightarrow \infty } H(a) = 0$$. Hence, $$H(a) \ge 0 $$ for all $$a>0$$ and (57) is established. For any $$a > 0 $$, set $$\tilde{a}$$ such that

59 $$\begin{aligned} \int _{0}^{a} \bar{F}_{T}(t) dt = \int _{0}^{\tilde{a}} \bar{F}_{\tilde{T}}(t) dt. \end{aligned}$$

It follows from (57) that $\tilde{a} > a$. Let $B \sim {\mathsf{Ber}}(a/\tilde{a})$. Let *V* have distribution (31) and let $\tilde{V}$ have the distribution (31) with *a* replaced by $\tilde{a}$ and *T* replaced by $\tilde{T}$. The survival functions of $\tilde{V}$ and *BV* are

60 $$\begin{aligned} \bar{F}_{\tilde{V}}(w)&= \left\{ \begin{array}{ll} 1, & w \in [0,\bar{F}_{\tilde{T}}(\tilde{a})) \\ \frac{1}{\tilde{a}} \bar{F}^{-1}_{\tilde{T}}(w), & w \in [\bar{F}_{\tilde{T}}(\tilde{a}),1) \\ 0, & w > 1, \end{array} \right. \end{aligned}$$

and

61 $$\begin{aligned} \bar{F}_{\tilde{B}\tilde{V}}(w)&= \left\{ \begin{array}{ll} \frac{a}{\tilde{a}}, & w \in [0,\bar{F}_{T}(a)) \\ \frac{1}{\tilde{a}} \bar{F}^{-1}_{T}(w), & w \in [\bar{F}_{T}(a),1) \\ 0, & w > 1. \end{array} \right. \end{aligned}$$

Since $\bar{F}_{T} - \bar{F}_{\tilde{T}}$ has a single sign change from positive to negative, it follows that $\bar{F}_{\tilde{V}} - \bar{F}_{BV}$ also has a single sign change from positive to negative. Hence, $V \le _{\mathrm{cx}} \tilde{B}\tilde{V}$ (Shaked and Shanthikumar 2007, Theorem 3.A.44). Applying Lemma 8 and Shaked and Shanthikumar (2007, Theorem 3.A.21) together shows $X(N, \tilde{V}) \le _{\mathrm{cx}} X(N,B V)$. From Theorem 1, $\tilde{M}(\tilde{a}) {\mathop {=}\limits ^{d}} \sum _{k=1}^{\Lambda (\tilde{a})} \tilde{X}_{k}$ and $M(a) {\mathop {=}\limits ^{d}} \sum _{k=1}^{\Lambda (a)} X_{k}$, where $\tilde{X}_{k} {\mathop {=}\limits ^{d}} X(N, \tilde{V})$ and $X_{k} {\mathop {=}\limits ^{d}} X(N,V)$. Let $B_{1}, B_{2},\ldots$ be a sequence of independent ${\mathsf{Ber}}(a/\tilde{a})$ random variables that are also independent of $X_{1},X_{2},\ldots$ By construction $BX(N,V) = X(N,BV)$. From the thinning property of the Poisson process, $M(a) {\mathop {=}\limits ^{d}} \sum _{k=1}^{\Lambda (\tilde{a})} B_{k} X_{k}$. As the convex order is closed under random sums, we see $\tilde{M}(\tilde{a}) \le _{\mathrm{cx}} M(a)$. Letting $a\rightarrow \infty$ and noting that the convex order is closed under weak limits, we see $\tilde{M}(\infty ) \le _{\mathrm{cx}} M(\infty )$. $\square$

That increasing variability in the parasite lifetimes decreases parasite aggregation seems counter-intuitive. However, if we consider the extreme case where the parasite lifetimes are constant, then we see that at any given age the host will either have all or none of the hosts from a previous infectious contact. Therefore, it is natural to expect this to lead to the greatest parasite aggregation. On the other hand, greater variability in the parasite lifetimes effectively spreads out when parasites die, leading to less parasite aggregation.

### Asymptotic normality

As noted previously, when the host’s parasite load to converges to a normal distribution, the Gini and Petra indices can each be approximate by a constant multiple of the coefficient of variation as indicated by the limits (28) and (30). The final result shows that when the rate of infectious contacts in the Tallis-Leyton model tends to infinity, the distribution of the host’s parasite load converges to a normal distribution.

#### Theorem 10

Suppose there exists positive constants $\epsilon,\ \delta$ and *C* such that

62 $$\begin{aligned} \left| G_{N}(1 + \omega ) - \left( 1 + G_{N}'(1) \omega + \frac{1}{2} G_{N}''(1) \omega ^{2} \right) \right| \le C \left| \omega \right| ^{2+\epsilon } \end{aligned}$$

for all $\omega \in \mathbb {C}$ such that $|\omega| < \delta$. Then

63 $$\begin{aligned} \lim _{\lambda \rightarrow \infty } \frac{M(a) - \mu (a)}{\sigma (a)} {\mathop {=}\limits ^{d}} {\mathsf{N}}(0,1). \end{aligned}$$

#### Proof

The characteristic function of *M*(*a*) is $G_{M}(e^{i\omega };a)$. We aim to show that

64 $$\begin{aligned} \lim _{\lambda \rightarrow \infty } e^{-i\tfrac{\omega \mu (a)}{\sigma (a)}} G_{M}(e^{i \tfrac{\omega }{\sigma (a)}};a) = \exp \left( - \tfrac{1}{2} \omega ^{2}\right). \end{aligned}$$

The result then follows by Lévy’s convergence theorem. Define

65 $$\begin{aligned} R_{N}(\omega ) = G_{N}(1 + \omega ) - \left( 1 + G_{N}'(1) \omega + \frac{1}{2} G_{N}''(1) w^{2} \right). \end{aligned}$$

For non-negative integers *n* and real *x* define

66 $$\begin{aligned} R_{n}(x) = e^{ix} - \sum _{k=0}^{n} \frac{(ix)^{k}}{k!}. \end{aligned}$$

Then $$|R_{0}(x)| \le \min (2,|x|)$$ and

67 $$\begin{aligned} \left| R_{n}(x) \right| \le \min \left( \frac{2|x|^{n}}{n!}, \frac{|x|^{n+1}}{(n+1)!} \right). \end{aligned}$$

(Williams 1991, pg 183). Note that

68 $$\begin{aligned} e^{-i\tfrac{\omega \mu (a)}{\sigma (a)}} G_{M}(e^{i \tfrac{\omega }{\sigma (a)}};a) = \exp \left( \lambda \int ^a_0 \left[ G_{N}(1 + \bar{F}_{T}(a-s) (e^{i \tfrac{\omega }{\sigma (a)}}-1) ) - 1 \right] ds - i \tfrac{\omega \mu (a)}{\sigma (a)} \right). \end{aligned}$$

From the expressions for $$R_N $$ and $$\mu (a)$$,

69 $$\begin{aligned}&\lambda \int ^a_0 \left[ G_{N}(1 + \bar{F}_{T}(a-s) (e^{i \tfrac{\omega }{\sigma (a)}}-1) ) - 1 \right] ds - i \tfrac{\omega \mu (a)}{\sigma (a)} \nonumber \\&\quad = \lambda G_{N}'(1) \left( \int ^a_0 \bar{F}_{T}(a-s) ds\right) R_{1} \left( \frac{\omega }{\sigma (a)} \right) + \frac{\lambda }{2} G_{N}''(1) \left( \int ^a_0\bar{F}_{T}^2(a-s) ds\right) R_{0} \left( \frac{\omega }{\sigma (a)} \right) ^2\nonumber \\&\quad + \lambda \int _0^a R_{N}\left( \bar{F}_{T}(a-s) (e^{i\tfrac{\omega }{\sigma (a)}} -1 ) \right) ds \end{aligned}$$

From the expression for $\sigma ^{2}(a)$ and the fact that

70 $$\begin{aligned} R_{1}(x)^2 + x^{2} = R_2(2x) - 2R_2(x), \end{aligned}$$

we obtain

71 $$\begin{aligned}&\lambda \int ^a_0 \left[ G_{N}(1 + \bar{F}_{T}(a-s) (e^{i \tfrac{\omega }{\sigma (a)}}-1) ) - 1 \right] ds - i \tfrac{\omega \mu (a)}{\sigma (a)} \nonumber \\&\quad = -\frac{\omega ^{2}}{2} + \lambda G_{N}'(1) \left( \int ^a_0 \bar{F}_{T}(a-s) ds\right) R_{2} \left( \frac{\omega }{\sigma (a)} \right) \nonumber \\&+ \frac{\lambda }{2} G_{N}''(1) \left( \int ^a_0\bar{F}_{T}^2(a-s) ds\right) \left( R_{2} \left( \frac{2\omega }{\sigma (a)} \right) - 2 R_{2} \left( \frac{\omega }{\sigma (a)} \right) \right) \nonumber \\&\quad + \lambda \int _0^a R_{N}\left( \bar{F}_{T}(a-s) (e^{i\tfrac{\omega }{\sigma (a)}} -1 ) \right) ds \end{aligned}$$

Using the bound (67) and the fact that $\sigma ^{2}(a) \propto \lambda$, we see

72 $$\begin{aligned} \lim _{\lambda \rightarrow \infty } \lambda G_{N}'(1) \left( \int ^a_0 \bar{F}_{T}(a-s) ds\right) R_{2} \left( \frac{\omega }{\sigma (a)} \right) = 0 \end{aligned}$$

and

73 $$\begin{aligned} \lim _{\lambda \rightarrow \infty } \frac{\lambda }{2} G_{N}''(1) \left( \int ^a_0\bar{F}_{T}^2(a-s) ds\right) \left( R_{2} \left( \frac{2\omega }{\sigma (a)} \right) - 2 R_{2} \left( \frac{\omega }{\sigma (a)} \right) \right) = 0. \end{aligned}$$

Finally, using $\left| R_{N}(\omega ) \right| \le C|\omega|^{2+\epsilon }$ together with the bound (67) and the fact that $\sigma ^{2}(a) \propto \lambda$, we see

74 $$\begin{aligned} \lim _{\lambda \rightarrow \infty } \lambda \int _0^a R_{N}\left( \bar{F}_{T}(a-s) (e^{i\tfrac{\omega }{\sigma (a)}} -1 ) \right) ds = 0. \end{aligned}$$

Hence, the limit (64) holds. $\square$

**Fig. 4:**
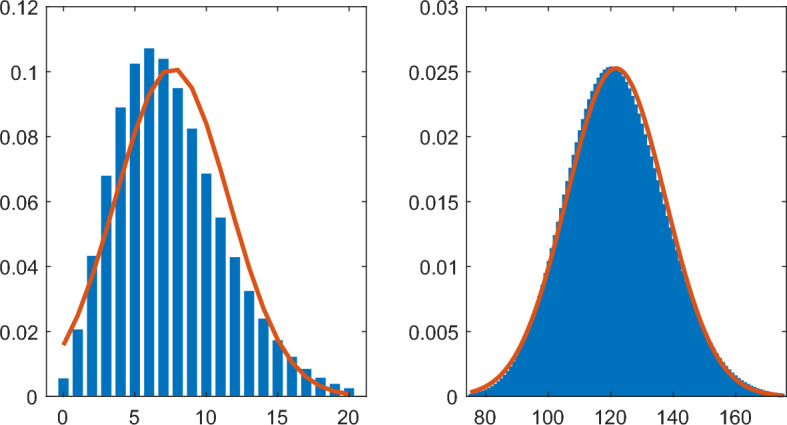
Probability mass function (blue bars) and approximating normal probability density function (red line) for a host aged 3 in the Tallis-Leyton model with $N \sim {\mathsf{NB}}(1,1)$, $T \sim {\mathsf{Exp}}(1)$, and $$\lambda = 8\ $$ (left) and $$\lambda = 128\ $$ (right)

**Fig. 5:**
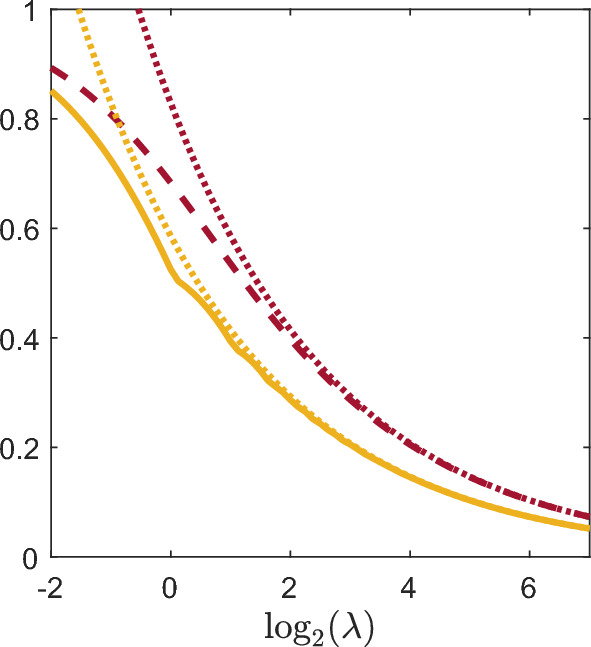
Gini index (purple dashed line) and Pietra index (yellow line) together with the asymptotic normal approximations (dotted lines) for a host aged 3 in the Tallis-Leyton model with $N \sim {\mathsf{NB}}(1,1)$ and $T \sim {\mathsf{Exp}}(1)$

Figure 4 compares the probability mass function of the host’s parasite load, *M*(*a*), in the Tallis-Leyton model with the probability density function of the approximating normal distribution. The Tallis-Leyton model used a host aged $a=3$, number of parasites (*N*) entering the host during an infectious having a ${\mathsf{NB}}(1,1)$ distribution, and parasite lifetimes (*T*) having an ${\mathsf{Exp}}(1)$ distribution. When the rate of infectious contact $\lambda = 8$, the probability mass function still shows some right skewness. The normal approximation in this instance places a non-negligible probability on values less than zero. When $\lambda = 128$, the probability mass function is very close to symmetric and the normal distribution provides a good approximation. Figure 5 shows the Gini and Pietra indices together with the approximations based on the limits (28) and (30). In this instance the approximations of the Gini and Pietra indices appear reasonably accurate even for $\lambda$ as small as 2 where the normal approximation is poor.

## Discussion

This study examined how variation in *M*(*a*), the parasite load of age *a* hosts, in the Tallis-Leyton model is affected by the host age, the rate of infectious contacts ($\lambda$), the distribution of the number of parasites (*N*) entering the host during an infectious contact, and the distribution of parasite lifetimes (*T*). Variation in the parasite load was quantified by several aggregation metrics. While there are many aggregation measures used in the parasitology literature, this study focused on measures related to the Lorenz ordering of distributions, specifically the coefficient of variation, the Gini index, Pietra index, and $1 - {{\text {prevalence}}}$. The Lorenz based measures of aggregation all decrease together if variation in the distribution decreases in the sense of the Lorenz order and are constrained by equality (24) and inequality (25). Furthermore, when the parasite load has approximately a normal distribution the Gini and Pietra indices can each be approximate by a constant multiple of the coefficient of variation.

The analysis showed that an increase in the rate of infectious contacts or an increase in the host age results in a decrease in the aggregation of parasite load using the Lorenz based measures. These results are perhaps not surprising in light of the behaviour of the Poisson distribution, which decreases in the Lorenz order as the mean increases. It might also be expected that increased variability in the the number of parasites entering the host during an infectious contact results in increased aggregation of parasite load using the Lorenz based measures. However, that increased variation in the parasite lifetimes decreases parasite aggregation in the limit as host age tends to infinity seems counter-intuitive. This result can be understood as variability in parasite lifetimes spreads out when parasites die and hence results in less variable parasite loads.

Although only four measure of aggregation based on the Lorenz order were explicitly mentioned in this study, these results extend to any other index respecting the Lorenz order. On the other hand, measures of aggregation not based on the Lorenz order may behave differently. For example, the variance-to-mean ratio is not affected by changes to the rate of infectious contacts. Also, if the number of parasites entering the host during an infectious contact has a ${\mathsf{NB}}(m,k)$ distribution, then an increase in *m* results in an increase in the variance-to-mean ratio, but the Lorenz based measures decrease.

Unfortunately, the population dynamics of parasites are often more complicated than what is represented in the Tallis-Leyton model. Some parasites need multiple hosts to complete its life cycle. Once a parasite finds a host it may be subject to intraspecific and interspecific competition for resources. Furthermore, parasites often interact with the host either by stimulating an immune response from the host or by increasing the host’s mortality rate.

Isham (1995) proposed a simple stochastic model that incorporates parasite induced host mortality. In Isham’s model, the host acquires parasites following the same dynamics as the Tallis-Leyton model and parasite lifetimes are assumed exponentially distributed. The important difference in Isham’s model is that each parasite present in the host increases the host’s death rate by a fixed amount $\alpha$. A complete analysis of Isham’s model in terms of the Lorenz order is beyond the scope of this paper. In a special case, however, we see that parasite induced host mortality increases aggregation of the parasite distribution in the sense of the Lorenz order. When the number of parasites that enter the host at an infectious contact follows a geometric distribution, an explicit expression for the limiting distribution is possible. Specifically, if $N \sim {\mathsf{NB}}(m,1)$, then

75 $$\begin{aligned} M(\infty ) \sim {\mathsf{NB}}\left( \frac{\lambda m}{ \mathbb {E} \left[ T\right] + \alpha + \alpha m}, \frac{\lambda }{ \mathbb {E} \left[ T\right] + \alpha + \alpha m}\right). \end{aligned}$$

As the negative binomial distribution is decreasing in Lorenz order in both mean and *k*, it follows that indices respecting the Lorenz order are increasing in the parasite induced host mortality rate. In contrast, the variance-to-mean ratio is $$1 + m $$ so it is not affected by the parasite induced mortality.

A complete examination Isham’s model in terms of the Lorenz order may prove challenging. Even computing the Gini and Pietra indices may present difficulties since they require absolute moments, which are often not easily evaluated. In that case, the coefficient of variation may prove useful since it respects the Lorenz order, is easily evaluated, and can be used to approximate the Gini and Pietra indices when the distribution is approximately normal.

## Data Availability

The code used to generate the figures in this paper are publicly available on Zenodo (McVinish 2025).
